# Estetrol: A New Choice for Contraception

**DOI:** 10.3390/jcm10235625

**Published:** 2021-11-29

**Authors:** Franca Fruzzetti, Tiziana Fidecicchi, Maria Magdalena Montt Guevara, Tommaso Simoncini

**Affiliations:** Department of Obstetrics and Gynecology, Pisa University Hospital, 56126 Pisa, Italy; t.fidecicchi@gmail.com (T.F.); magdalena.montt@gmail.com (M.M.M.G.); tommaso.simoncini@unipi.it (T.S.)

**Keywords:** combined hormonal contraception, estetrol, metabolism, SHBG, cycle control, hemostasis

## Abstract

Estetrol (E4) is a natural estrogenic steroid that is normally produced by human fetal liver. Recent research has demonstrated that it is a potent, orally bioavailable, natural selective estrogen receptor modulator; it has a moderate affinity for both human estrogen receptor alpha (ERα) and ERβ, with a preference for ERα. Clinical studies have demonstrated possible use as an estrogen in combined oral contraceptives (COC). COCs containing E4 and drospirenone (DRSP) showed a high acceptability, tolerability, and user satisfaction also when compared to COCs containing ethinylestradiol (EE). E4/DRSP effectively inhibits ovulation, with a similar effect on endometrium thickness than that of EE-containing COCs. Low doses (15 mg) of E4 with DRSP (3 mg) showed promising results in term of bleeding pattern and cycle control, also when compared to other COCs containing synthetic estrogens. Moreover, the association has limited effects on serum lipids, liver, SHBG levels, and carbohydrate metabolism. This combination also could drive a lower risk of venous thromboembolism than EE-containing COCs. In this review, we will summarize the actual knowledge about the new E4-containing contraceptive. Further large-scale studies in the full target population are needed to provide more insights into the cardiovascular safety profile and user satisfaction of E4/DRSP.

## 1. Introduction

Estetrol (E4) is a human natural estrogen which was discovered in 1965 in urines of pregnant women [1,2]. It is only produced during human pregnancy and it reaches the maternal circulation through the placenta [3,4]. Human maternal plasma levels increase during pregnancy, reaching high concentrations towards the end of gestation (≥1 ng/mL). Fetal plasma levels have been reported to be nearly 20 times higher than maternal plasma levels at parturition [5]. After delivery, blood levels of E4 become rapidly undetectable [6,7]. It is of interest that E4 is not produced by other species tested so far (mice, rat, and rabbit). The role as a marker of fetal health have been studied for many years but no correlation was found [7,8]. Even now the physiological significance of E4 in pregnancy is unknown.

In this review we summarize the actual knowledge about E4, focusing the attention on its new application for hormonal contraception. 

## 2. Biosynthesis and Pharmacological Properties

**Figure 1:**
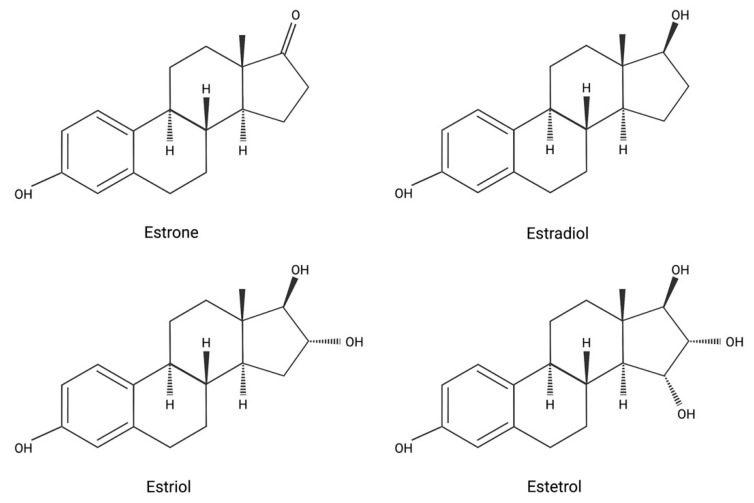
Molecular structure of different estrogens. Created with BioRender.com (4 September 2021).

Its chemical structure is the 15-hydroxyethryol or the oestra-1,3,5(10)-trien-3,15-16,17-tethrol: the molecule has 4-OH groups, so it’s also called with the name E4 (Figure 1). 

E4 is synthesized during pregnancy from estradiol (E2) and estriol (E3) by two fetal liver enzymes through hydroxylation. These two enzymes are *15α* and *16α-hydroxylase* and they are expressed only during fetal age [4].

E4 is extensively metabolized or inactivated by human hepatocytes in vitro, producing metabolites by direct glucuronidation of the D-ring and direct sulfation at an unconfirmed site. E4 is mainly excreted in the urine rather than through the biliary route, and it is the terminal product of its own pathway: there are not secondary reactions that could produce E3, E2, or estrone (E1) [9,10]. 

E4 displays moderate protein binding (close to 50%) in human plasma. E4 does not bind to human sex hormone-binding globulin (SHBG) [11]. It is equally distributed between plasma and blood cells in human blood. E4 has a high oral bioavailability (90%) and an important long half-life in humans, with an average of 28–32 h half-life, which is about two-fold longer than E2 [9]. In contrast to E2, E4 does not show clinically relevant inhibiting or inducing interactions with cytochrome P450 liver enzymes or with other molecules [11,12].

In the 1970s and 1980s different studies demonstrated that E4 has a weaker estrogenic activity than E2, E3, and tamoxifen: this activity has been shown in uterus [5,7,8]. In fact, study shows that E4 has a low to moderate affinity for both human estrogen receptor α (ERα) and β (ERβ) with a 4/5-fold preference for ERα [10]. Based on this relatively low receptor binding affinity compared to E2, E4 was originally thought to be a weak estrogen [12,13]. 

Recent studies indicate that E4 is an estrogen with a distinctive profile of ERα activation. E4 activates the nuclear ERα, but it is an antagonist of the membrane ERα, in contrast to other estrogens [14,15,16]. Based on its pharmacological profile, E4 can be classified as the first Natural Estrogen with Selective Action in Tissues (NEST) [17]. NEST activities of E4 are the consequence of its unique dual role.

### Effects on Different Tissues

E4 may display different effects on different tissues due to its agonist or antagonist activity on ERs. 

In well validated and predictive rat models, E4 behaves as an estrogen agonist in all tissues investigated, i.e., bone, vagina, myometrium, endometrium, and brain, and it is effective in inhibiting ovulation by reducing follicle stimulating hormone (FSH) and luteinizing hormone (LH) plasma concentrations [8,18,19]. A study reported a beneficial effect of E4 on bone, through the analysis of different markers like: bone mineral density (BMD), mineralization of vertebral bodies from L3 to L5, strength against biomechanical damages and level of serum osteocalcin [18]. Another set of study was done on CNS, suggesting a neuroprotective role of E4 [19,20]. It was observed that in ovariectomized rats treated with E4 the expression of allopregnanolone and β-endorphin in serum was increased and different cerebral areas like frontal cortex, hippocampus, and pituitary gland were induced. The expression of those neurosteroids are reduced when E4 and E2 are given together [19,20].

Most beneficial effects of E4 on the vascular system have been ascribed to the activation of the membrane ERα of vascular endothelial cells, including enhancement of nitric oxide (NO) production, vasodilation, and prevention of atherosclerosis, of neointimal proliferation, and of hypertension [8]. 

On breast tumor tissue it acts as an estrogen antagonist in the presence of E2 [21,22]. The estrogen-antagonistic effect of E4 in the breast has been further supported by a recent pre-clinical study that has been performed in women with breast cancer, finding that E4 reduces breast cancer cells proliferation [21,23,24,25,26]. These features could suggest a future role of E4 as a selective estrogen receptor modulator (SERM), but with less adverse effect than tamoxifen (hot flushes, nausea, hypertension, thromboembolic events, endometrial hyperplasia) [24,27,28]. However, in 2008 a study showed that E4 has a weak proliferative activity on mammary tumoral MCF-7 cells [27] and surprisingly it acts as an estrogen antagonist on rat breast DMBA model cells, where it prevents the development of new breast tumors and stimulates the regression of pre-existing ones [28,29]. 

Some of the principal physiological role and properties of E4 on different tissues like bone, uterus, vagina, breast, CNS, and ovaries are shown in Table 1.

These features are very important because they mean that E4 could be a useful and safe molecule in hormonal therapy. In this field, an important application of E4 is for combined oral contraceptives (COCs). It holds promises for the safety and tolerability of COCs containing E4.

## 3. New Combined Hormonal Contraception with Estetrol

COCs traditionally contain an estrogen and a progestin component. Estrogens are useful to stabilize the endometrium, to regulate menstrual bleeding and to reduce follicle development. The most used estrogen is ethinylestradiol (EE), variously combined with different progestins, but it has an impact on liver function and endothelium that can produce rare cases of venous or arterial thrombotic complications. Since 2009 COCs containing E2 has been developed to reduce this effect. E4 may represent another valid option for COCs, with many advantages linked to its dual effect on receptors. 

### 3.1. Ovulation and Cycle Control: How to Reach the Best Patients’ Satisfaction

The ovulation inhibitory potency of E4 was first studied in preclinical models, followed by clinical trials in women (Table 2). 

Coelingh Bennink et al. in 2008 [30] studied the effectiveness of E4 as an ovulation inhibitor in regularly cycling rats compared to EE. Rats were treated orally twice daily for four consecutive days with E4 (0.03, 0.1, 0.3, 1.0, or 3.0 mg/kg), EE (0.0003, 0.001, 0.003, 0.01, or 0.03 mg/kg) or vehicle control. Ovulation was significantly inhibited with a dose of 0.3 mg/kg of E4 twice daily and above and with 0.03 mg/kg of EE twice daily. In a second experiment they also administered 2.0 mg/kg of E4 once daily or divided in two doses of 1.0 mg/kg: this second option was able to inhibit ovulation in all treated rats, while the single-dose administration acted in half of them. EE resulted to be 18 times more potent than E4 [30]. 

The ability to suppress LH and FSH production in female humans was studied in early post-menopause women. E4 showed a profound central inhibitory and dose dependent effect on LH and FSH in post-menopause women [33]. After a single-dose administration, a clear dose-dependent inhibition of LH levels and a profound inhibition of FSH levels over 48 h after 100 mg of E4 (lasting over 7 days) was observed [9].

A phase II dose-finding pilot study evaluated the efficacy of different dosages of E4 combined with levonorgestrel (LNG) or drospirenone (DRSP) in suppressing the pituitary-ovarian axis and ovulation in healthy premenopausal women [31]. E4 combined with DRSP (5 or 10 mg E4 + 3 mg DRSP) or LNG (5, 10 or 20 mg E4 + 0.15 mg LNG) in a 24/4-day regimen was compared to EE 20 mcg + 3 mg DRSP, all of them administered for three consecutive cycles. The highest suppression of ovarian activity was observed in the 20 mg E4/LNG group and was very similar to that observed with EE/DRSP. However, there were no ovulations during the treatment cycles in all treatment groups, showing the efficacy of all the combinations of E4. Endometrial thickness was also reduced similarly during treatment in all treatment groups. 

The first post-treatment ovulation occurred approximately 17 days after the last treatment day in the E4/DRSP groups, and 21 days after the last active treatment in the E4/LNG and EE/DRSP groups: this period was comparable to the duration of a normal follicular phase, confirming adequate ovarian suppression during treatment. In conclusion, a dosage above 10 mg/day of E4 with DRSP or LNG demonstrated to be a promising combination for contraception [31]. 

Another phase II study aimed to assess bleeding patterns and cycle control of E4 containing COCs in a 24/4-day regimen, using a COC containing estradiol valerate (E2V) and dienogest (DNG) as a reference [32]. E4 15 and 20 mg/DRSP 3 mg, E4 20 mg/LNG 0.15 mg and E2V/DNG were compared after six treatment cycles. The frequency of unscheduled bleeding/spotting was lower in the E4/DRSP groups compared to the other treatment groups: by cycle 6, the frequency varied between 33.8% in the group using 15 mg of E4 + DRSP and 47.8% in the E2V/DNG group, with increasing intensity of unscheduled bleeding over time in the E2V/DNG group. For E4/DRSP, the frequency of absence of withdrawal bleeding was 3.5 (15 mg E4) to 3.8% (20 mg E4) at cycle 6. In the E4/LNG groups, the frequencies were 14.0–18.5%, and for E2V/DNG it was 27.1%. Only 8.9% of subjects in the group using 15 mg of E4 + DRSP discontinued prematurely. In conclusion, the 15 mg E4/DRSP combination has been shown to be the most efficacious in terms of bleeding pattern and cycle control, compared with the other combinations investigated [32]. The largest proportion of treatment satisfaction was reported for 15 mg E4/DRSP (73.1%) compared to 20 mg E4/DRSP and 15 or 20 mg E4/LNG. Well-being with E4/DRSP combinations was statistically significantly better than with E4/LNG combinations and the administration of 15 mg E4/DRSP favors a good weight control, with 36.7% of women losing 2 kg or more after 6 months of treatment [36]. 

After these dose-finding studies, phase III clinical trials using the combination E4 15 mg/DRSP 3 mg were performed. Two studies are available by now, for a total of around 3400 women enrolled and followed for a period of 13 cycles. In the first study conducted in 1864 women aged 16 to 50 years old from North America, the PI was 2.65 in women aged 16 to 35 (54% of the undesired pregnancies due to method failure, with a method-failure PI of 1.43) [35]. In the second study, conducted in 1553 women from Europe and Russia between the ages of 18 and 50 years old, less pregnancies were reported, and the Pearl Index (PI) was 0.47 in the group aged 18 to 35 and 0.41 in the whole group, with a method-failure PI of 0.25 in the whole group. This was considered a sufficiently low value for an oral contraceptive. Scheduled bleeding occurred in 91.9–94.4% of participants per cycle. Scheduled bleeding and/or spotting days remained stable throughout the study with a median duration of 4 to 5 days. Unscheduled bleeding and/or spotting episodes after Cycle 1 occurred in 19.2% of women in Cycle 2 and decreased to 12.8% of women in Cycle 11. Among these episodes over all cycles, 71.8% were spotting-only episodes, 22.7% were mixed bleeding/spotting and 5.4% were bleeding-only [34]. Overall, a COC with E4 15 mg/DRSP 3 mg was considered effective at preventing unwanted pregnancies with a satisfactory bleeding pattern control. 

### 3.2. Hemostatic effect

Historically, EE containing COCs demonstrated their efficacy and safety, with a satisfactory bleeding pattern. However, their impact on liver function and vascular endothelium could produce rare cardiovascular thrombotic complications that could limit their use in a subgroup of women. The use of androgenic progestins and the substitution of EE with E2 can modulate and reduce this risk [41,42]. E4 represents a promising option to be used for COCs. From in vitro to in vivo studies investigated its influence on hemostatic parameters.

In an in vitro study on human umbilical vein endothelial cells, the effects of E4 on fibrinolytic system and whether it could influence the ability of endothelial cells to migrate were studied [43]. Expression of plasminogen-activator inhibitor-1 (PAI-1), urokinase-type plasminogen activator (u-PA), and tissue plasminogen activator (t-PA) proteins were all increased by E4 in a dose-dependent manner, although E4 was less effective than equimolar amounts of E2. Moreover, endothelial cell migration capacity was increased by E4 treatment. So, it was concluded that E4 could regulate the fibrinolytic protein system in endothelial cells, with potential implications for the local control of blood clotting and for vascular remodeling [43]. 

Mouse models were also used to test E4 impact on arterial and venous thrombosis. It increased mouse tail bleeding time, it protected from both arterial and venous thrombosis, and it induced a resistance against acute thromboembolism. Ex vivo flow-based adhesion studies conducted in whole blood under arterial flow conditions on a collagen matrix showed that E4 treatment also reduced platelet adhesion [15]. 

In human studies using the new COC containing E4, more insights about this issue were given (Table 2). Kluft et al. [37] evaluated the effects of 3 mg DRSP in combination with 5 or 10 mg of E4 compared to a preparation containing EE 20 μg/DRSP 3 mg. Over three months, the E4-containing preparations had a much lower impact. Both E4 combinations reduced D-dimer level with no effect on antithrombin, protein S activity or activated protein C resistance, and the 5 mg E4/DRSP combination also decreased prothrombin fragment 1 + 2. It can be stated that E4/DRSP have a considerably lower hepatic and vascular estrogenicity than EE/DRSP [37]. 

In addition, Douxfils et al. [38] demonstrated that after six months of treatment E4 15 mg/DRSP 3 mg caused similar or smaller changes in procoagulant, anticoagulant, and fibrinolytic parameters than EE/LNG, while the difference with EE/DRSP was more pronounced [38].

### 3.3. Estetrol, Metabolism, and Cardiovascular Risk

One of the earliest manifestations of atherosclerosis is the dysfunction of the vascular endothelium, caused by one or more “insults” to the endothelium. This leads to a decrease in release of the vasodilator, nitric oxide, and an increase in production of the vasoconstrictor, endothelin-1. Although endothelial dysfunction occurs early in the atherosclerotic process, it continues throughout the progression of the disease. A second major consequence of damage to the endothelium is the accumulation of inflammatory cells in the vascular wall, that causes the oxidation and accumulation of low-density lipoproteins (LDL). This activates a vicious cycle which leads to the atheroma [44].

Estrogens are known to have a vasculoprotective action, which has been demonstrated clearly in animal models of early atheroma: E2 has been shown to strongly prevent fatty streak deposition in monkeys, rabbits, and mice [45]. They have a general cardioprotective effect: they prevent atherosclerosis [46,47], they reduce hypertensive effects of ovariectomy [48], they reduce age- and hypertension-related arterial stiffening [49], they increase the production of nitric oxide [50], they accelerate endothelial reparation processes [51], they prevent intimal post-traumatic hyperplasia [52].

E4 demonstrated to have similar vasculoprotective action in mice thanks to its binding with ERα, even if with less potence than E2 [53,54,55]. Moreover, it has positive effects on metabolic parameters. A study in post-menopausal women treated with different doses of E4 (2 mg, 10 mg, 20 mg, or 40 mg) for 28 days showed that a lowering effect on LDL was accompanied with an increase in HDL and no or minimal changes in triglycerides; all the effects were dose-dependent [56].

E4 associations with different progestins were studied to evaluate their metabolic effects, in view of the formulation of new COCs (Table 2). Mawet et al. [39] made a dose-finding study in healthy normally ovulating women aged 18–35 years. They administered six different treatments in six groups of women for three consecutive cycles in a 24/4-day regimen: 5 mg or 10 mg of E4 with 3 mg of DRSP; 5 mg, 10 mg, or 20 mg E4 with 0.15 mg LNG; 0.02 mg EE with 3 mg DRSP as comparator. E4-containing COCs caused minor effects on lipoproteins and triglycerides compared to the EE/DRSP group [39]. In addition Klipping et al. [40] found similar results. The combination of 15 mg of E4 and 3 mg of DRSP had minimal impact on lipid parameters. The largest effect was observed for triglycerides, that showed a 24% increase after treatment; however, this increase was less compared to EE/LNG (+28%) and EE/DRSP (+65.5%) [40]. 

Phase III studies will better address this issue, giving information on a large population. However, phase II results show that E4/DRSP combination is substantially neutral on lipid parameters.

### 3.4. Effects on the Synthesis of Other Liver Proteins

E4-containing COCs have a limited effect on liver function (Table 2). 

SHBG is a carrier protein produced in the liver. It binds estrogens and testosterone, and its levels may be used as a surrogate to evaluate the steroid effects on the liver. SHBG may be considered as a marker for estrogenicity of a contraceptive preparation and possibly for the risk of venous thrombosis [57,58]. In general, estrogens can cause a dose-related increase of SHBG levels, while progestogens induce a decrease of SHBG levels, depending on the type and the dose of the progestogen used. Thus, the combination of the estrogenic effect of the estrogen contained in the hormonal contraceptive and of the antiestrogenic effect of the progestogen used (effect that is higher with androgenic progestins) determines the total estrogenicity of that hormonal contraceptive. 

SHBG plasma levels decreased with E4 (5, 10, or 20 mg)/LNG, while showed a dose-dependent slight increase with 5 or 10 mg E4/DRSP (+7.9–44.5%). This increase is considerably less than with EE/DRSP [39]. In addition Klipping et al. [40] found a similar variation of SHBG: E4 15 mg/DRSP 3 mg caused an increase of 87.15% of SHBG levels at cycle 6, still less than EE/LNG and EE/DRSP [40]. Other studies confirmed the little influence of this COC on SHBG production [37,38]. Other liver proteins were studied, too. Angiotensinogen changes showed a profile similar to SHBG [45,46,59]. C-reactive protein, cortisol binding globulin, thyroxin binding globulin, and ceruloplasmin showed slight changes with E4/DRSP [39,40], confirming the limited estrogenic effect of E4 on liver also when combined to antiandrogenic progestins.

### 3.5. Bones and Breast: Safety of E4-Containing COCs

Studies about the effects of the new proposed COCs with E4 on tissues like bone and breast are still scant. 

Even if some data about the preventive effect on bone loss of E4 alone are already available [18,59], by now the only available information about effects on bone of E4-containing COCs is given by the study by Mawet et al. [39] (Table 2). A balance between bone resorption and bone formation maintains the regulation of bone mineral density. This study did not detect any imbalances after treatment with E4/DRSP, E4/LNG or the comparator EE/DRSP in serum osteocalcin (a marker of bone formation) and C-telopeptide (a marker of bone degradation). This may be indicative of a positive influence on bone turnover in young post-adolescent women, similarly to EE- or E2-containing COCs [39].

Similarly, very little is known about the effects of E4/DRSP combinations on breast. Recently, a study on breast cancer was published [60]. It shows that E4 combined with or without progesterone or DRSP promotes neither breast cancer development nor metastatic dissemination in three different models of breast cancer when used at a therapeutic dose for hormone replacement therapy or COC [60]. The results may suggest that the use of E4 in contraception could further limit the already minimal possible effect of other hormonal contraceptives on breast cancer risk. Nevertheless, at the present it is not possible to extrapolate clinical data about this issue.

## 4. Conclusions

In conclusion, pharmacological properties of E4 make it a useful molecule for hormonal therapies and contraception. By now, some phase II and phase III studies gave promising results using the combination of E4 15 mg/DRSP 3 mg: this COCs showed a good contraceptive effect and cycle control, with a neutral metabolic effect. However, still very little is known about the effects of this new combination on breast and bone. Post-marketing studies are needed to consolidate the available data and to explore all the possible side effects and risks for bone, breast, and cardiovascular system of a long-term use of E4-containing pills compared to the well-known EE- and E2-containing combinations.

## Figures and Tables

**Table 1:** Effects of estetrol on the estrogen receptor α (ERα) and β (ERβ) on different tissues: brain, bone, female genital system, and breast.

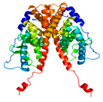	Low affinity for estrogen receptors [12,13] Higher affinity for ERα than ERβ [10] A natural SERM [24,27,28]
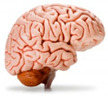	Neuroprotective effects [19,20] Inhibition of LH and FSH secretion [8,18,19]
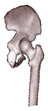	Prevention of bone demineralization [18] Increase bone mineral density [18]
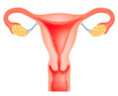	Inhibition of ovulation [30] Uterine growth and epithelial proliferation [16,31,32]
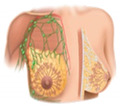	Less known Estrogenic and anti-estrogenic effects on horizontal migration and matrix invasion of ER+ T47-D [19,30,33]

**Table 2:** Phase II and phase III studies about new estetrol (E4)-containing combined oral contraceptives (COCs) are summarized. Studies are divided according to the outcomes studied.

Outcomes.	Study	E4 Combinations Tested	Comparators	Results
Ovulation inhibition and cycle control	Phase II Duijkers et al., 2015 [31]	5 or 10 mg E4 + 3 mg DRSP 5, 10, or 20 mg E4 + 0.15 mg LNG 24 + 4 regimen	0.02 mg EE + 3 mg DRSP 24 + 4 regimen	No ovulation in any treatment group. Ovarian activity inhibition proportional to E4 dosage (20 mg E4/LNG like EE/DRSP) Endometrial thickness similarly suppressed in all groups Post-treatment ovulation occurred in all patients in the first month.
	Phase II Apter et al., 2016 [32]	15 or 20 mg E4 + 3 mg DRSP 15 or 20 mg E4 + 0.15 mg LNG 24 + 4 regimen	4-phasic commercial packaging of E2V/DNG 26 + 2 regimen	Lowest frequency of unscheduled bleeding and/or spotting and absence of withdrawal bleeding in the 15 mg E4/DRSP group
	Phase III Gemzell-Danielsson et al., 2021 [34]	15 mg E4 + 3 mg DRSP 24 + 4 regimen	/	Method-failure PI: 0.29 pregnancies/100 woman-years Scheduled bleeding occurred in 91.9–94.4% of women per cycle Unscheduled bleeding/spotting episodes decreased in the first 6 cycles and remained stable thereafter (<16%)
	Phase III Creinin et al., 2021 [35]	15 mg E4 + 3 mg DRSP 24 + 4 regimen	/	Method-failure PI in 16–35 years old women: 1.43 pregnancies/100 woman-years Scheduled bleeding occurred in 82.9 to 87.0% of women per cycle Unscheduled bleeding decreased in the first 4 cycles and remained stable thereafter (15.5% to 19.2%)
Treatment satisfaction	Phase II Apter et al., 2017 [36]	15 or 20 mg E4 + 3 mg DRSP 15 or 20 mg E4 + 0.15 mg LNG 24 + 4 regimen	4-phasic commercial packaging of E2V/DNG 26 + 2 regimen	The largest proportion of treatment satisfaction in the 15 mg E4/DRSP group; the lowest in the 15 mg/LNG group Well-being with E4/DRSP: better than with E4/LNG Proportion of women with a 2 kg or more weight loss: the highest with 15 mg E4/DRSP
Hemostatic effect	Phase II Kluft et al., 2017 [37]	5 or 10 mg E4 + 3 mg DRSP 24 + 4 regimen	0.02 mg EE + 3 mg DRSP 24 + 4 regimen	E4/DRSP no or minor effect on markers of coagulation inhibition; they were reduced by EE/DRSP thus promoting coagulation E4/DRSP did not increase D-dimer levels, unlike EE/DRSP
	Phase II Douxfils et al., 2020 [38]	15 mg E4 + 3 mg DRSP 24 + 4 regimen	0.02 mg EE + 3 mg DRSP 24 + 4 regimen 0.03 mg EE + 0.15 mg LNG 21 + 7 regimen	Changes in hemostasis parameters after treatment with 6 cycles of E4/DRSP were smaller or like those observed for EE/LNG Similar, but more pronounced changes were also observed versus EE/DRSP
Metabolic effect	Phase II Mawet et al., 2015 [39]	5 or 10 mg E4 + 3 mg DRSP 5, 10, or 20 mg E4 + 0.15 mg LNG 24 + 4 regimen	0.02 mg EE + 3 mg DRSP 24 + 4 regimen	Minor effects on lipid levels (HDL- and LDL- cholesterol) with E4/DRSP and E4/LNG Triglycerides levels: reduced with E4/LNG, the same with E4/DRSP and increased with EE/DRSP
	Phase II Klipping et al., 2021 [40]	15 mg E4 + 3 mg DRSP 24 + 4 regimen	0.03 mg EE + 0.15 mg LNG 21 + 7 regimen 0.02 mg EE + 3 mg DRSP 24 + 4 regimen	E4/DRSP had minimal impact on lipid parameters The largest effect was observed for triglycerides, still less than EE/LNG and EE/DRSP E4/DRSP: no effect on carbohydrate metabolism
SHBG and other liver proteins	Phase II Klipping et al., 2021 [40]	15 mg E4 + 3 mg DRSP 24 + 4 regimen	0.03 mg EE + 0.15 mg LNG 21 + 7 regimen 0.02 mg EE + 3 mg DRSP 24 + 4 regimen	Liver proteins, except CRP, increased in all groups, but the effect for angiotensinogen and SHBG was less pronounced with E4/DRSP compared to EE/LNG and EE/DRSP
	Phase II Mawet et al., 2015 [39]	5 or 10 mg E4 + 3 mg DRSP 5, 10, or 20 mg E4 + 0.15 mg LNG 24 + 4 regimen	0.02 mg EE + 3 mg DRSP 24 + 4 regimen	SHBG and other liver proteins were minimally or not affected by E4/LNG and E4/DRSP Changes of SHBG and other liver proteins were more marked in the EE/DRSP group
	Phase II Kluft et al., 2017 [37]	5 or 10 mg E4 + 3 mg DRSP 24 + 4 regimen	0.02 mg EE + 3 mg DRSP 24 + 4 regimen	SHBG and angiotensinogen increase with 10 mg E4/DRSP was 15%−20% that of EE/DRSP 5 or 10 mg E4/DRSP had nearly no effect on SHBG and minor effect on angiotensinogen
	Phase II Douxfils et al., 2020 [38]	15 mg E4 + 3 mg DRSP 24 + 4 regimen	0.02 mg EE + 3 mg DRSP 24 + 4 regimen 0.03 mg EE + 0.15 mg LNG 21 + 7 regimen	Changes in SHBG values for E4/DRSP, EE/LNG and EE/DRSP were +55%, +74% and +251%, respectively
Bones: safety study	Phase II Mawet et al., 2015 [39]	5 or 10 mg E4 + 3 mg DRSP 5, 10, or 20 mg E4 + 0.15 mg LNG 24 + 4 regimen	0.02 mg EE + 3 mg DRSP 24 + 4 regimen	E4 groups: dose-related decrease of biomarkers of bone resorption (C-telopeptide) and bone formation (osteocalcin) Decreased bone turnover in all E4 and EE combinations: it is indicative of a similar positive influence on bone turnover in young post-adolescent women

CRP, C-reactive protein; DNG, dienogest; DRSP, drospirenone; E2V, estradiol valerate; EE, ethinylestradiol; HDL, high density lipoprotein; LDL, low density lipoprotein; LNG, levonorgestrel; PI, pearl index; SHBG, sex hormone binding globulin.
